# Bullosis Diabeticorum: Rare Presentation in a Common Disease

**DOI:** 10.1155/2014/862912

**Published:** 2014-11-18

**Authors:** Vineet Gupta, Neha Gulati, Jaya Bahl, Jaswinder Bajwa, Naveen Dhawan

**Affiliations:** ^1^Department of Medicine, University of California San Diego (UCSD), 200 West Arbor Drive, MC 8485, San Diego, CA 92103, USA; ^2^Department of Medicine, Morristown Medical Center, Morristown, NJ 07960, USA; ^3^Florida International University (FIU), Miami, FL 33174, USA; ^4^Nova Southeastern University Health Sciences Division, Fort-Lauderdale-Davie, FL 33314, USA

## Abstract

A 27-year-old African American male presented with a sudden onset of blisters. He had a past medical history of uncontrolled diabetes mellitus type I, diabetic vasculopathy, and neuropathy. The physical examination revealed nonerythematous skin denudations on both elbows and lateral aspect of arm bilaterally. Investigations which included skin biopsies confirmed the diagnosis of bullosis diabeticorum. The bullae were treated with hydrotherapy and healed with no complications in 4 weeks. We present this case to illustrate the rare occurrence of diabetic bulla in a diabetic patient especially with poor glycemic control. The case is also a reminder of the importance of diabetes screening in nondiabetic patients who are diagnosed with diabetic bulla.

## 1. Background

Diabetes mellitus is associated with cutaneous manifestations including diabetic thick skin, acanthosis nigricans, necrobiosis lipoidica diabeticorum, and diabetic dermopathy in about one-third of patients [1–3].Bullosis diabeticorum is a spontaneous, noninflammatory, and blistering condition, that is, uniquely affects patients with diabetes mellitus. We present a case of bullosis diabeticorum in a patient with a history of diabetes mellitus type 1 who presented with a sudden onset of blisters that were diagnosed as diabetic bullae.

## 2. Case Presentation

**Figure 1:**
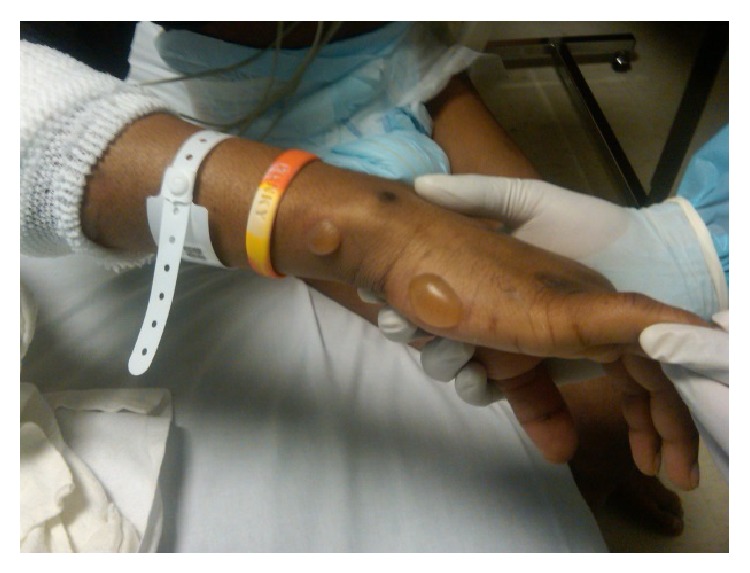
Tense serous fluid filled bullae.

**Figure 2:**
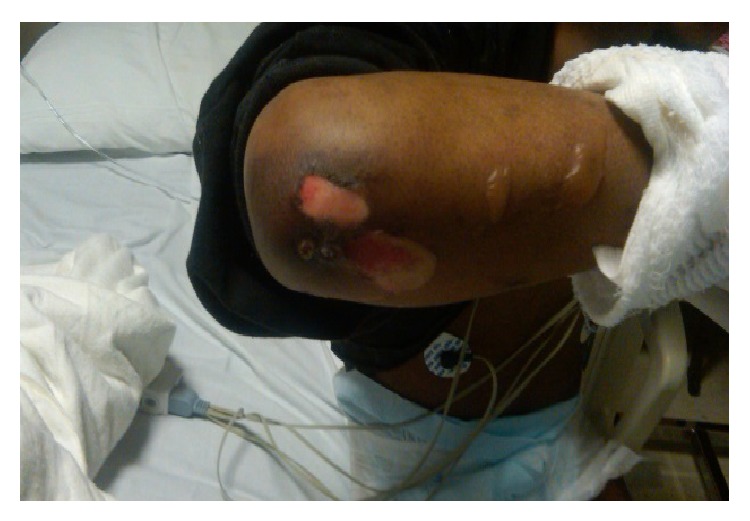
Skin ulceration due to bullae rupture at the right elbow.

**Figure 3:**
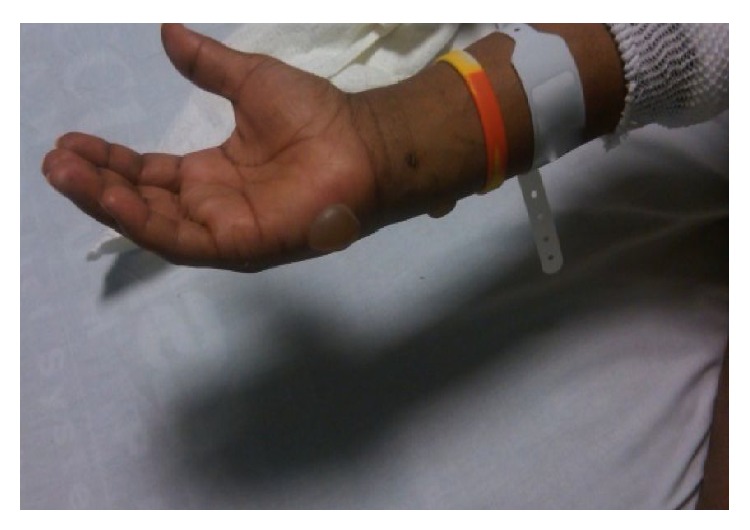
Bulla on the palmar aspect of right hand.

A 27-year-old African American male with past medical history significant for uncontrolled diabetes mellitus type I, diabetic vasculopathy, neuropathy, and medical noncompliance presented to our hospital with sudden onset of blisters on elbows bilaterally. Apparently, the patient slept on the floor and woke up 6 hours later with the skin lesions. The patient denied any recent trauma, travel, exposure to chemicals, intoxication, insect bite, or any constitutional symptoms. Physical examination was notable for nonerythematous discontinuous stage II skin ulceration 6 × 4 cm on left elbow on flexural aspect and 3 × 4 and 2 × 2 cm on right elbow joint along with multiple clear fluid filled blisters on flexural and lateral aspect of arm bilaterally (Figures 1, 2, and 3). Lesions were moderately painful.

## 3. Investigations

Lesional biopsies were taken for H and E staining; perilesional skin was sent for direct immunofluorescence (DIF) for IgG, IgM, and IgA to exclude clinically similar conditions such as bullous pemphigoid, epidermolysis bullosa acquisita, and porphyrias. Complete blood count was unremarkable.

## 4. Differential Diagnosis

Differential diagnosis included other immune bullous disorders such as bullous pemphigoid, epidermolysis bullosa acquisita, traumatic blisters, bullae due to drug reactions, insect bites, and bullous SLE.

## 5. Treatment

Patient underwent hydrotherapy and silvadene dressing changes daily by the plastic surgery team. He was also given elbow pads to avert any trauma to the lesions.

## 6. Outcome and Follow-Up

The biopsy was remarkable for subepidermal bulla extending as an intraepidermal cleft with sparse nonspecific infiltrate containing occasional neutrophils and rare eosinophils. DIF was negative for IgG, IgM, and IgA. The lesions healed without complications over subsequent 4 weeks with skin care. Patient was discharged with close follow-up for tight glycemic control.

## 7. Discussion

Bullosis diabeticorum (BD) or diabetic bulla is a spontaneous, recurrent, noninflammatory, and blistering condition usually affecting acral and distal skin of lower extremities [1–3]. The blisters are usually large and asymmetrical in shape [4]. These serous fluid filled tense bullae (sized few mm to cm) may even sometimes be hemorrhagic [5]. The condition occurs in about 0.5% of diabetics in the USA [1]. They are seen in patients from 17 to 80 years of age and are more frequent in adult men suffering from long standing uncontrolled diabetes with peripheral neuropathy [6, 7]. BD shows a higher frequency in males, with a male-to-female ratio of 2 : 1 [1, 8]. There are only about 100 cases describing BD in the worldwide literature [9].

The etiology of BD has not been elucidated [10]. While some have postulated that trauma in diabetic patients may be the cause, this may be unlikely due to the occurrence of several lesions over different regions of the body [1]. Also, many bullae are reported that occurred in patients without any apparent antecedent trauma. Interestingly, it can also be seen in patients with nephropathy, microangiopathy, and regulatory disorders of carbohydrates, calcium, and magnesium [7, 11]. For some patients, blisters have been found in relation to UV exposure. ESRD patients can have mildly elevated porphyrin levels that can contribute to blister formation.

The diagnosis of BD entails punch biopsies and subsequent histopathologic examination [12]. The histologic features of bullosis diabeticorum are not very specific. Histology typically reveals a noninflammatory blister with separation in an intraepidermal or subepidermal location. Anchoring fibrils and hemidesmosomes tend to be decreased. Caterpillar bodies which are often found in patients with porphyria can also be seen. Immunofluorescence staining would typically be negative for IgM, IgG, IgA, or C3; in the presence of characteristic presentation, the diagnosis of BD can be then confirmed [12].

Healing is usually spontaneous in a few weeks but close monitoring for any secondary bacterial infection or hemorrhage is warranted [7]. One study that followed 25 patients with BD over a 3-year period described the median healing time for patients to be 2.5 months [5]. It is recommended that the blisters should be left intact to serve as a sterile dressing preventing secondary infection. Some treatments include the aspiration of blisters using a small bore needle to prevent accidental rupture. Topical antibiotics can be applied to prevent infections and petroleum jelly can be used to alleviate pain or discomfort [13]. The condition can frequently recur and is rarely complicated by osteomyelitis [4, 14]. The lesions on the feet can turn into chronic ulcers followed by necrosis and infection [5]. Tissue necrosis may necessitate debridement and tissue grafting.

Importantly, while they are usually found in patients with diabetes, diabetic bulla may also appear in prediabetic patients [12]. One of the reasons the condition may be underdiagnosed is that it may heal spontaneously in patients who do not seek medical attention. In addition, it is possible that clinicians who treat diabetic patients may miss the presence of BD during routine visits. An awareness of BD may help clinicians to take prompt action and improve patient comfort while averting secondary infections. This case underscores the importance of considering BD as possibility while managing diabetic patients.

## 8. Learning Points

- Diabetic bulla is a rare disorder associated with long term diabetes mellitus which is poorly recognized among physicians and may be largely undiagnosed.
- A high index of suspicion is warranted in diagnosing and instituting appropriate therapy for averting secondary infections or ulcers.
- The diagnosis of bullosis diabeticorum in a nondiabetic patient should prompt screening for diabetes.
